# Myopia and its associated factors among pregnant women at health institutions in Gondar District, Northwest Ethiopia: A multi-center cross-sectional study

**DOI:** 10.3389/fgwh.2022.1078557

**Published:** 2023-01-16

**Authors:** Mengistie Diress, Mihret Getnet, Yonas Akalu, Baye Dagnew, Yitayeh Belsti, Yibeltal Yismaw Gela, Dagmawi Chilot, Daniel Gashaneh Belay, Desalegn Anmut Bitew, Bewuketu Terefe, Amare Belete Getahun

**Affiliations:** ^1^ Department of Human Physiology, University of Gondar, Gondar, Ethiopia; ^2^ Department of Epidemiology and Biostatistics, Institute of Public Health, University of Gondar, Gondar, Ethiopia; ^3^ Department of Human Anatomy, School of Medicine, University of Gondar, Gondar, Ethiopia; ^4^ Department of Reproductive Health, Institute of Public Health, University of Gondar, Gondar, Ethiopia; ^5^ Department of Community Health Nursing, School of Nursing, University of Gondar, Gondar, Ethiopia; ^6^ Department of Anesthesia, School of Medicine, University of Gondar, Gondar, Ethiopia

**Keywords:** myopia, pregnancy, Ethiopia, prevalance, women

## Abstract

**Background:**

Myopia is the most common form of uncorrected refractive error with a growing burden worldwide. It is the principal complaint of most women during pregnancy. Although myopia has led to several consequences across the standard life of pregnant women, there is no previous study in Ethiopia regarding this topic. Thus, this study determined the prevalence of myopia and identifies its associated factors among pregnant women attending antenatal care units at governmental health institutions in Gondar City District, Northwest Ethiopia.

**Methods:**

An institution-based cross-sectional study design was conducted from 08 February to 08 April 2021. From the selected health centres, study participants were recruited by systematic random sampling technique. A pre-tested, structured-interviewer-administered questionnaire consisting of socio-demographic variables, obstetric and clinical-related variables was used to collect the required data. Non-cycloplegic refraction was performed using trial lenses, trial frames, and retinoscopy in a semi-dark examination room. EpiData 3 and STATA 14 were used for data entry and statistical analysis respectively. Both bivariable and multivariable binary logistic regression analyses were executed to identify associated factors of myopia. Variables with a *p*-value ≤0.05 in the multivariable logistic regression analysis were declared as statistically significant with myopia. Model fitness was checked by Hosmer and Lemeshow goodness of test (at *p* > 0.05)

**Results:**

A total of four-hundred and twenty-three pregnant women participated with a 100% response rate in this study. The overall prevalence of myopia among pregnant women was 26.48% (95% CI: 22.48–30.91). Eighty-Eight (20.81%) and Eighty-Four (19.85%) of the study participants had myopia in their right and left eyes respectively. The prevalence of myopia was significantly associated with age (AOR = 1.17; 95% CI: 1.09–1.28), the third trimester of gestation (AOR = 2.05, 95% CI: 1.08–3.90), multi & grand multipara (AOR = 3.15; 95% CI: 1.59–6.25), and history of contraceptive use (AOR = 3.30; 95% CI: 1. 50–7.28).

**Conclusion:**

The finding of our study shows that there is a higher prevalence of myopia among pregnant women in our study area. Further prospective analytical studies regarding visual systems among pregnant women, particularly as a result of pregnancy, are strongly recommended.

## Introduction

Myopia, (defined as “the spherical equivalent of objective refraction is ≤ – 0.50 diopter in either eye or both”), is the most common form of uncorrected refractive error. Myopia is the chief cause of visual impairments across the globe irrespective of age and sex, affecting about 30% of the world's population (1, 2). Nowadays, Myopia is a frightening pandemic refractive problem affecting about 2.5 billion people worldwide (3). As the recent systematic review and meta-analysis has suggested, about 34% of the global population became myopic by 2020 and half (49.8%) of the world's population may be affected by myopia by 2050 (4).

According to different studies across the world, the prevalence of myopia among young adults in many developed western countries is 20%–40% and 5%–10% in less developed countries (5). It is 33.3% among the European population (6), 80%–90% in East and Southeast Asia (5), 39.1% in France (7), 26.9% in the United Kingdom (8), and 14.08% in Africa (4).

According to studies carried out in different countries of the world, adult females are highly prone to develop myopia than males (9–12). In Europe, 42.3% of women are affected by nearsightedness (6). Myopia is detected in 27.5% of females according to a study in Israel (13). The prevalence of myopia among Chinese women is about 45.4% (8). The study in Saudi Arabia estimated that the prevalence of myopia among women is 18.1% (14). The prevalence rate of myopia among female medical students based on another study in Saudi Arabia is 34.6% (15). Based on the study in Ethiopia, the prevalence of myopia among female school-age children is about 27% while only 12% among male students (16).

Metabolic and hormonal changes during pregnancy can upset the normal visual functions of the women's eyes. Myopia is the principal complaint of most women during pregnancy. This problem is due to either physiological changes during pregnancy or exacerbations of pre-existing medical conditions (17, 18). Most myopic changes that happened during pregnancy are transient but occasionally, they may lead to permanent complications which will interfere with the usual health of the women (17, 19). Based on the study in Iran, myopia is observed in 11.77% of pregnant women which is more aggravated in the third trimester of gestation (18). A study in India revealed that 65% of pregnant women have myopia (20). The prevalence of myopia among pregnant women was reported as 77.50%, based on a study in South India (21). A study in Nigeria shows that myopia is the most prevalent type of refractive error among pregnant women which accounts for 57% (17). According to a recent institution-based cross-sectional study in Ethiopia, 35.66% of pregnant women have refractive errors (22).

The global burden of myopia is increasing over time and influences the quality of life of individuals, by way of poor vision (low vision and blindness), low productivity, and social interactions (1, 4, 23). Myopia is also a cause of retinal degenerative changes (retinal detachment) which may lead to intra- and post-partial ophthalmological complications during pregnancy (24). It can also increase the risk of social loneliness and depression, lead to the inability to perform tasks alone, increase the risk of fall-related injuries, and sexual violence and abuse (25).

Based on previous studies in the world, refractive error most commonly myopia, is associated with the age of study participants (25–28), residence (25, 29, 30), educational status (2, 31–33), occupation/job (30, 34, 35), gestational age (GA) (17, 27, 36), maternal parity (27, 34, 37), diabetes mellitus (38, 39), gestational diabetes mellitus (GDM) (26, 34), pre-existing hypertension (34, 40), pregnancy-induced hypertension (PIH) (26, 34), family history of vision problem/myopia (28, 30, 41), prolonged use of smartphones and computers (42–44), medication history (26, 45, 46), history of contraceptive use (26, 47), and spent more time for sleeping at night (28).

Although myopia has led to several consequences across the quality of life of pregnant women, there is no previous study in Ethiopia regarding this topic. Thus, this study aimed to determine the prevalence of myopia and associated factors among pregnant women attending antenatal care units at selected governmental health institutions in Gondar district, Northwest Ethiopia**.** Information on the prevalence of myopia among pregnant women can help clinicians and policymakers to design appropriate prevention strategies.

## Methods and materials

### Study design, setting, and population

An institution-based cross-sectional study design was conducted from 08 February to 08 April 2021. The study was conducted at selected governmental health institutions in Gondar District. Gondar is a historical city in Ethiopia located 727 km far from the capital city, Addis Ababa in the Northwest direction. It has 12 sub-cities with 12 urban and 10 rural kebeles. In Gondar district, there are eight health centres and one teaching referral hospital providing antennal care (ANC) services for about 41,000 pregnant women annually. This study was conducted among pregnant women of the 15–49 age group. All pregnant women who visited ANC services of the selected health institutions were included in the study whereas; those with congenital eye problems and eye trauma during the study period were excluded.

### Sample size determination and sampling procedure

**Figure 1:**
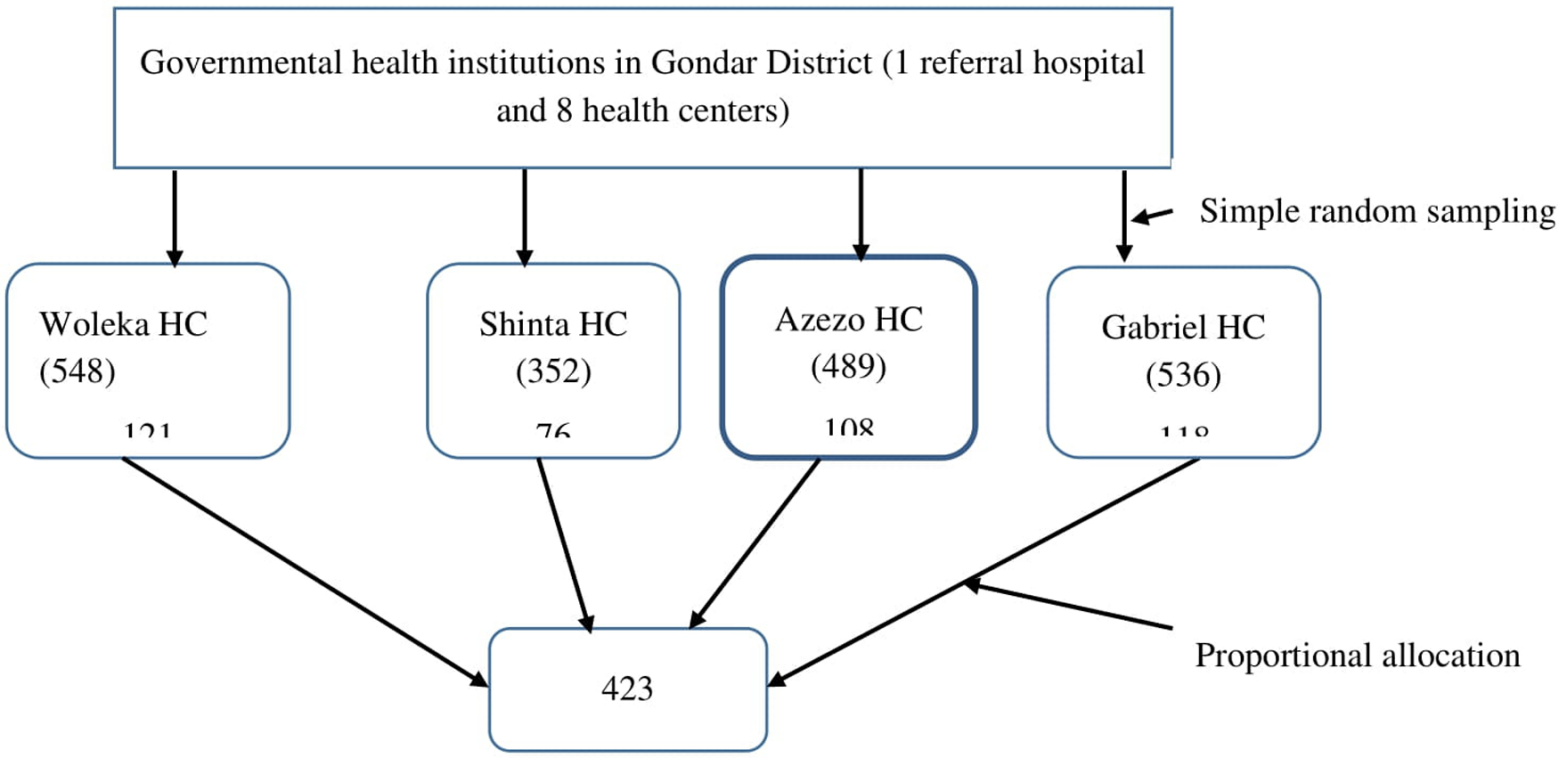
Selection of pregnant women (sample size) visiting ANC services at governmental health centers in Gondar District, Gondar, Ethiopia, 2021.

Single population proportion formula was used to calculate the required sample size. A 0.5 proportion of the population with myopia has been taken to estimate the minimum sample size because there was no study in the same study area. 5% margin of error, 95% confidence interval, and 10% non-response rates were also considered to calculate the sample size. Hence, the total sample size became 423. A simple random sampling method was used to select health institutions for the study. Four health centres from the district were randomly selected by lottery methods. From the selected health centres, study participants were recruited by systematic random sampling technique. To improve the representativeness of the sample size to the source population, the proportional allocation was performed for each health institution (Figure 1).

### Study variables

The dependent variable was myopia, which was dichotomized as “yes” or “no”. We classified the study participants as “yes” (myopic) if the spherical equivalent of objective refraction is ≤–0.50 diopter in either eye or both and unless otherwise, normal (no) if the spherical equivalent of objective refraction is >–0.50 diopter in either eye or both.

The independent variables were age, residence, occupation, educational level**,** parity, gestational age, history of DM, GDM, history of HTN, PIH (preeclampsia and eclampsia), history of medication, regular use of smartphones and computers or watching TV, history of contraceptive use, sleep disturbance, and family history of vision problem.

### Operational definitions

**Myopia:** the spherical equivalent of objective refraction is ≤–0.50 diopter in either eye or both. The severity of myopia is categorized as Mild myopia: spherical equivalent = −0.50 to −2.99 D; Moderate myopia: spherical equivalent = −3.00 to −6.00 D; High myopia: spherical equivalent greater than −6.00 D (2, 48, 49).

**Regular use of computers or television**: Reading or watching computers or television at least once a day for not less than 2 h (50).

**Regular use of smartphones**: Using smartphones at least once a day for more than 2 h (50).

**Sleep disturbance**: Sleeping time of ≤5 h/day or sleeping time of ≥9 h/day (51, 52).

**Medication History:** Taking anti-rheumatic, anti-psychiatric & anti-thrombotic drugs in the last 30 days.

### Data collection tools, procedures, and quality management

A pre-tested, structured-interviewer-administered questionnaire consisting of socio-demographic variables, obstetric and other clinical-related variables was used to collect the required data. Presenting visual acuity test was determined using Snellen's illiterate “E” chart in a well-illuminated room, at a distance of 6 meters from the chart. Non-cycloplegic refraction was performed for all study participants using trial lenses, trial frames, and retinoscopy in a semi-dark examination room. Data were collected by two BSc Midwives and two Optometrists. The training was given to the data collectors and the supervisor about the objectives of the study, data collection techniques and ethical issues. Strict supervision was undertaken during the process of data collection. The study participants had gotten counselling and a referral system depending on the ocular findings.

### Data processing and analysis procedure

The collected data were entered into EpiData 3.1 and exported into STATA 14 for statistical analysis. Descriptive measures like median, frequency and interquartile range (IQR) were calculated. Bi-variable binary logistic regression analysis was used to select the candidate variables for the final model. Those variables with a *p*-value of <0.2 in the bivariable binary logistic regression analysis were selected for multivariable binary logistic regression. Multivariable binary logistic regression analysis was executed to identify factors associated with myopia. The measure of association was defined by adjusted odds ratio (AOR) with a 95% confidence interval. In the final model, variables with a *p*-value ≤0.05 were declared as statistically associated with Myopia. Model fitness was checked by Hosmer and Lemeshow goodness of test (at *p* > 0.05) and multi-collinearity was tested by a variance inflation factor (VIF).

### Ethical approval and consent to participate

Prior to the study commencement, all the ethical issues were secured. Ethical clearance was gotten from the Institutional Review Board (IRB) of the University of Gondar with the reference number 1828/2012. A permission letter was obtained from Gondar district health office before data collection. This study was done in accordance with the relevant guidelines and regulations of the Declaration of Helsinki. After the study participants were adequately briefed about the study, written informed consent was taken from each study participant. Privacy and confidentiality of information were kept properly. Study participants who had moderate and high myopia at the time of data collection were referred to the Department of Ophthalmology at the University of Gondar Comprehensive Specialized Hospital for better diagnosis and management.

## Results

### Socio-demographic characteristics of the pregnant women

A total of four-hundred and twenty-three pregnant women participated with a 100% response rate in this study. The age range of pregnant women who participated in the study was from 16 to 46 years. The majority of the pregnant women (82.51%) were from urban residences. 37.12% of our study participants had a college or university level of education and 33.10% of them are housewives by occupation (Table 1).

**Table 1:** Socio-demographic characteristics of pregnant women attending ANC units at governmental health institutions in Gondar District, Northwest Ethiopia, 2021 (*n* = 423).

Variables	Frequency	Percentage (%)
**Age in years^c^**	Median = 27 (IQR = 7)	
**Religion**		
Christian	336	79.43
Muslim	87	20.57
**Residence**		
Urban	349	82.51
Rural	74	17.49
**Educational status**		
Cannot read & write	55	13.00
Primary	98	23.17
Secondary	113	26.71
College/University	157	37.12
**Occupation**		
Government employee	108	25.53
Private employee	85	20.09
Merchant	43	10.17
Housewife	140	33.10
Others^a^	47	11.11

^a^ Others = farmers, daily workers and unemployed.

^c^ continous Variable.

### Lifestyle, clinical, and obstetric-related characteristics

The majority of the study participants were nulli and primiparous (61.70%) and 64.78% of them were in third the trimester of gestation. Thirty-nine (9.22%) pregnant women had a history of vision problems (myopia). The majority of our study participants (61.70%) had used smartphones for more than 2 h per day. Two-hundred and forty (56.74%) of the study participants had a history of contraceptive use prior to their current pregnancy (Table 2).

**Table 2:** Lifestyle, clinical, and obstetric-related characteristics of pregnant women attending ANC units at governmental health institutions in Gondar District, Northwest Ethiopia, 2021 (*n* = 423).

Variables	Frequency	Percentage (%)
**Parity**		
Nulli & primi para	261	61.70
Multi & grand multipara	162	38.30
**Trimesters of gestation**		
1^st^ & 2^nd^ TM	149	35.22
3^rd^ TM	274	64.78
**History of DM**		
Yes	24	5.67
No	399	94.33
**Gestational diabetes mellitus**		
Yes	19	4.49
No	404	95.51
**History of hypertension**		
Yes	28	6.62
No	395	93.38
**Pregnancy-induced hypertension**		
Yes	29	6.86
No	394	93.14
**History of vision problem**		
Yes	39	9.22
No	384	90.78
**Regular use of smart phones**		
Yes	261	61.70
No	162	38.30
**Regular use of computer or television**		
Yes	204	48.23
No	219	51.77
**Medication history**		
Yes	46	10.87
No	377	89.13
**Contraceptive use**		
Yes	240	56.74
No	183	43.26
**Ever drink alcohol**		
Yes	289	68.32
No	134	31.89
**Currently drink alcohol**		
Yes	187	64.71
No	102	35.29
**Perceived stress level**		
Low	75	17.73
Moderate	315	74.47
High	33	7.80
**Sleep duration**		
Short	50	11.82
Optimal	268	63.36
Long	105	24.82

DM, diabetes mellitus; TM, trimester.

### Prevalence of myopia and its associated factors

**Figure 2:**
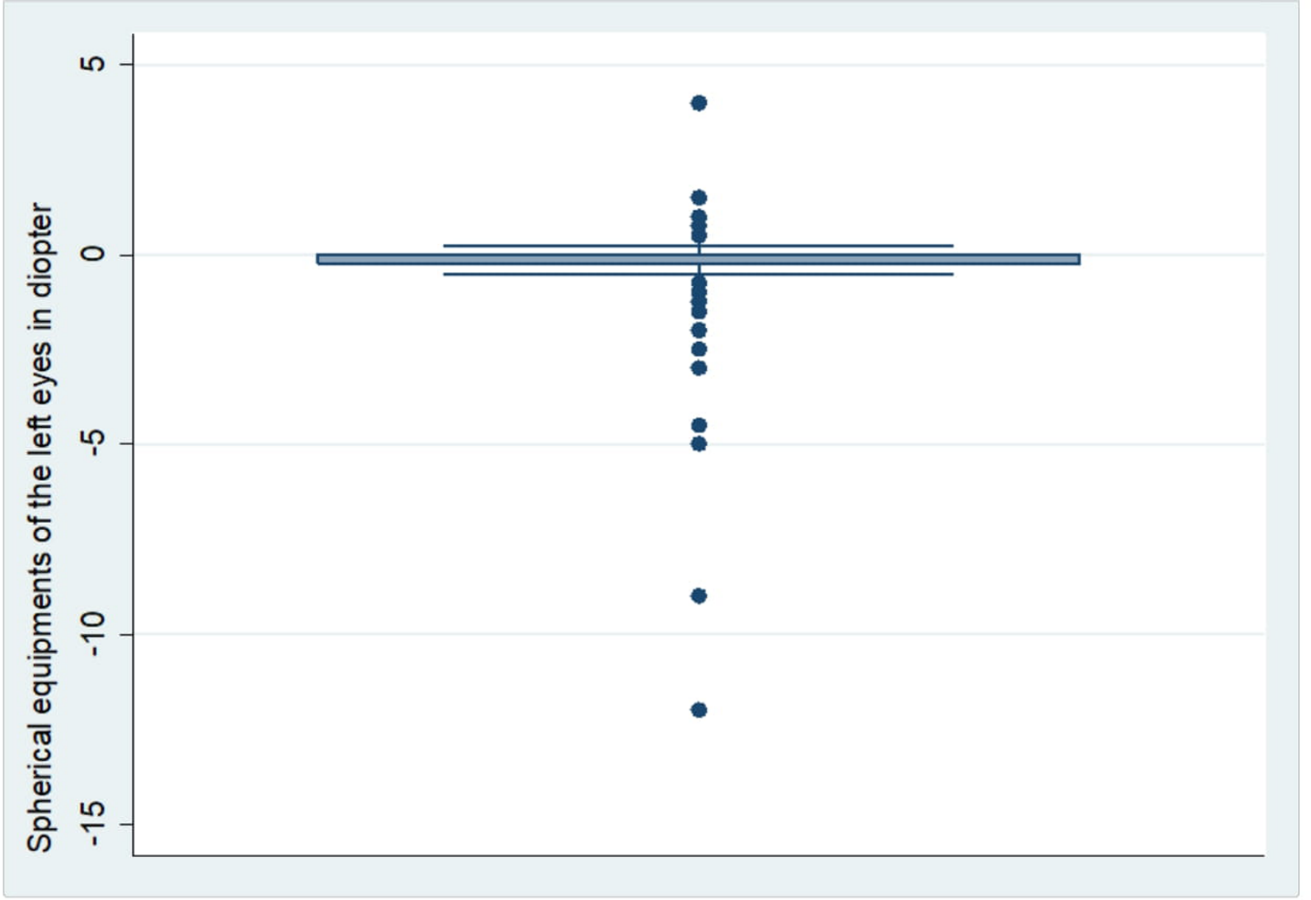
Distribution of spherical equivalent on the left eyes of pregnant women (*n* = 423).

**Figure 3:**
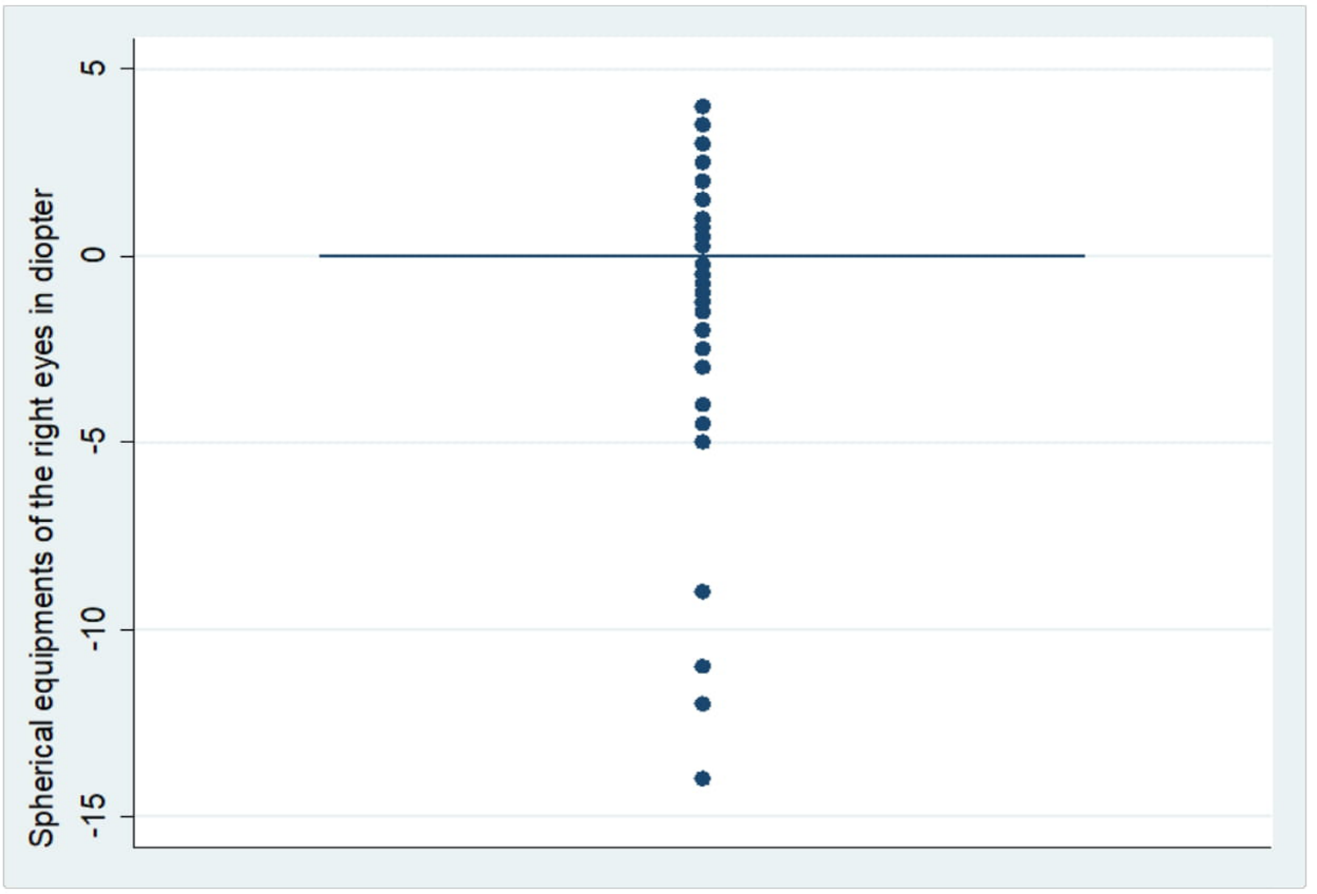
Distribution of spherical equivalent on the right eyes of pregnant women (*n *= 423).

In this study, the overall prevalence of myopia among pregnant women was 26.48% (95% CI: 22.48–30.91). Eighty-Eight (20.81%) and Eighty-Four (19.85%) of the study participants had myopia in their right (Rt) and left (Lt) eyes respectively. The spherical equivalent (SE) refractive error in the right and left eyes of the study participants ranged from −14.0D to +4.0D and −12.0D to +4.0D respectively. In both eyes, the majority of the study participants had 0.0D of SE (Two hundred fifty-five (60.28%) on their Rt eyes and Two hundred sixty-two (61.94%) on their Lt eyes) (Figures 2 & 3). The median spherical equivalent in both eyes (Rt & Lt) was 0.0 in our study. The spherical equivalent result showed that 61.46% of the women were emmetropic, 12.06% were hyperopic and the rest 26.48% were myopic (Table 3).

**Table 3:** Distribution of refractive error among pregnant women attending ANC units at governmental health institutions in Gondar District, Northwest Ethiopia, 2021 (*n* = 423).

RE	Frequency	Percentage (%)
Emmetropia	260	61.46
Hyperopia	51	12.06
Myopia	112	26.48
Total	423	100

Amongst all variables entered in to a binary logistic regression, age, residence, educational status, occupation, parity, gestational age, history of DM, GDM, History of HTN, PIH, family history of vision problem, history of contraceptive use, and history of medication were associated with myopia at *p*-value <0.2. However, in the final model, only age, parity, gestational age and history of contraceptive use were significantly associated with myopia at *p*-value ≤0.05.

The odds of developing myopia among study participants was increased by 1.17 times (AOR = 1.17; 95% CI: 1.09–1.28) for a unit increase in the age of pregnant women. Pregnant women who were in the third trimester of gestational age had 2.05 times (AOR = 2.05, 95% CI: 1.08–3.90) increased odds of myopia than those in the first and second trimesters of gestational age. Being multi & grand multiparous among pregnant women was 3.15 times (AOR = 3.15; 95% CI: 1.59–6.25) more likely to develop myopia than those who were nulli and primi parous. The odds of having myopia among pregnant women who had a history of contraceptive use prior to their current pregnancy was 3.3 times (AOR = 3.30; 95% CI: 1. 50–7.28) higher than the non-users (Table 4).

**Table 4:** Bivariable and multivariable logistic regression analysis of factors associated with myopia among pregnant women attending ANC units at governmental health institutions in Gondar District, Northwest Ethiopia, 2021 (*n* = 423).

Variables	Myopia		OR (95% CI)		*p*-value
	Yes, *N* (%)	No, *N* (%)	COR	AOR	
**Age (years)^c^**	112 (26.48)	311 (73.52)	1.27(1.20–1.34)	1.17(1.09–1.28)	**0** **.** **001** *
**Residence**					
Urban	84 (24.07)	265 (75.93)	1.00	1.00	
Rural	28 (37.84)	46 (62.16)	1.92 (1.13–3.26)	0.72 (0.30–1.72)	0.456
**Educational status**					
Can’t read & write	25 (45.45)	30 (54.55)	1.00	1.00	
Primary	21 (21.43)	77 (78.57)	0.33 (0.16–0.67)	0.74 (0.28–1.93)	0.534
Secondary	10 (8.85)	103 (91.15)	0.12 (0.05–0.27)	0.39 (0.12–1.23)	0.108
College/University	56 (35.67)	101 (64.33)	0.67 (0.36–1.24)	1.52 (0.50–4.62)	0.460
**Occupation**					
Government employee	38 (35.19)	70 (64.81)	2.17(1.23–3.85)	1.40(0.53–3.75)	0.498
Private employee	23 (27.06)	62 (72.94)	1.48(0.79–2.79)	1.35(0.55–3.31)	0.512
Merchant	9 (20.93)	34 (79.07)	1.06 (0.46–2.46)	1.18 (0.39–3.49)	0.766
House wife	28 (20.00)	112 (80.00)	1.00	1.00	
Others**	14 (29.79)	33 (70.21)	1.70(0.80–3.59)	2.49(0.83–7.49)	0.105
**Gestational age (weeks)**					
1^st^ & 2^nd^ TM	24 (16.11)	125 (83.89)	1.00	1.00	
3^rd^ TM	88 (32.12)	186 (67.88)	2.46 (1.49–4.08)	2.05 (1.08–3.90)*	**0** **.** **029** *
**Parity**					
Nulli & primi para	25 (9.58)	236 (90.42)	1.00	1.00	
Multi & grand multipara	87 (53.70)	75 (46.30)	10.95 (6.54–18.33)	3.15 (1.59–6.25)*	**0** **.** **001** *
**History of DM**					
Yes	11 (45.83)	13 (54.17)	2.50 (1.08–5.75)	2.41 (0.61–9.59)	0.210
No	101 (25.31)	298 (74.69)	1.00	1.00	
**Gestational DM**					
Yes	9 (47.37)	10 (52.63)	2.63 (1.04–6.65)	0.23 (0.05–1.12)	0.069
No	103 (25.50)	301 (74.50)	1.00	1.00	
**History of HTN**					
Yes	14 (50.50)	14 (50.50)	3.03 (1.40–6.58)	1.47 (0.45–4.76)	0.523
No	98 (24.81)	297 (75.19)	1.00	1.00	
**PIH**					
Yes	18 (62.07)	11 (37.93)	5.22 (2.38–11.45)	1.10 (0.36–3.37)	0.862
No	94 (23.86)	300 (76.14)	1.00	1.00	
**Family history of vision problem**					
Yes	16 (41.03)	23 (58.97)	2.09 (1.06–4.11)	2.36 (0.97–5.72)	0.057
No	96 (25.00)	288 (75.00)	1.00	1.00	
**History of contraceptive use**					
Yes	100 (41.67)	140 (58.33)	10.18 (5.30–0.72)	3.30 (1.50–7.28)	**0** **.** **003** *
No	12 (6.56)	171 (93.44)	1.00	1.00	
**History of medication**					
Yes	21 (45.65)	25 (54.35)	2.64 (1.41–4.94)	1.61 (0.66–3.95)	0.298
No	91 (24.14)	286 (75.86)	1.00	1.00	

AOR, adjusted odds ratio; c, continuous variable; CI, confidence interval; COR, crude odds ratio; TM, trimester; TV, television.

* *p*-value ≤0.05.

** Farmers, daily workers and unemployed.

## Discussion

Pregnancy is a normal physiological condition, which is often characterized by both physiological and pathological changes in all organ systems of the body including the visual system during pregnancy. Most of the changes during pregnancy are due to transient responses to the hormonal and metabolic modifications to take on the gestational product. There are also critical pathological complications that may persist after postpartum period in reproductive age women (53, 54). Refractive errors are the common types of ocular alterations among pregnant women, of which myopia is the largest variety but to the best of our knowledge, very little is known about the magnitude of myopia among pregnant women in Ethiopia. Thus, this study (the first of its kind in Ethiopia) tried to offer insight on the magnitude of myopia and its significant factors among pregnant women at health institutions in Ethiopia, the case of Gondar District governmental health institutions.

In our study, the overall prevalence of myopia among pregnant women was 26.48% (95% CI: 22.48–30.91) which is comparable with studies in Israel (27.5%) (13). However, our finding is lower than the studies conducted in India (65%) (20), South India (77.5%) (21), Nigeria 57% (17), and USA (25%–50%) (40). This discrepancy might as a result of the differences in study settings and study design. For instance, we applied institution based cross-sectional study while most of the previous studies used observational prospective studies. Another possible reason for the variation would be also cultural and socio-economic characteristics of the study population. The Ethiopian populations including women have the least exposure to potential risk factors like access to use digital devices and environmental hazards (industries) when compared to people of developed countries.

The prevalence of myopia among pregnant women in this study is higher than in other previous studies in Saudi Arabia (18.1%) (14), Iran (11.77%) (18), and South Africa (2.9%) (55). This variation might be attributable to the differences in the study population. Here, the study population in our study were only pregnant women whereas in the compared studies above are non-pregnant women.

Many previous studies in the world revealed that the prevalence of myopia is increased during pregnancy because of metabolic and hormonal changes (18, 26, 49, 54, 56). In the course of pregnancy, there is an increased level of estrogen and progesterone, which cause fluid retention in the cornea. This leads to corneal edema, thickness and curvature, and amplified lens thickness, which subsequently increases refractive power of the eye and end-up with myopia (18, 20, 21, 26, 54, 57). Myopia can be also associated with neuro-ophthalmic and other pre-existing conditions precipitated by gravidity (26).

A unit increase in years of maternal age was significantly associated with myopia which is in line with other studies in South India (58), United States of America (59), China (11, 48), and Sri Lanka (60). The increased likelihood of myopia with age might be due to an increased risk of age related diseases of the eye. With increasing of age, the nature and functions of the lens and cornea gradually decreases and strongly affects the normal focusing of the light at the retina (26).

Myopia was 2.05 times more likely to occur in the third trimester of GA of the women, which is consistent with other studies in South India (21), Turkey (61), Iran (62), and Nigeria (17). As reported by previous studies, the reason for this occurrence might be due to the metabolic and hormonal fluctuations because of gestational pressure, which may lead to corneal thickness and greater refractive power of the lens that finally brings about myopia among the pregnant women (21, 26, 63).

The odds of developing myopia was increased by 3.15 times among Multi & grand multiparous pregnant women than those who were nulli and primiparous. This result is in line with other previous study in South India (21) and China (34). This occasion is probably due to the repetitive ocular shifts because of hormonal influences on the subsequent gravidity of mothers who had higher number of parity. With increasing of parity, corneal edema, thickness, and curvature might be more elevated, which will upset the normal refraction power of the eyes.

The odds of having myopia among pregnant women who had history of contraceptive use before their current pregnancy was 3.3 times higher than the non-users. Our finding is similar with previous studies in India (64), Egypt (47), and Greece (65). This may be due to the fact that using contraceptives (oral and injectable) as a family planning method will cause corneal edema and an increase in the corneal thickness and curvature associated to the hormonal effects (estrogen and progesterone), which leads to myopia (66, 67).

A perfect response rate (100%**)** was the strength of our study. However, this study was cross-sectional, which did not measure the cause-effect relationship between independent variables and myopia. We did not also perform cycloplegic refraction test assuming that the procedure is exhaustive and its overall effect on the outcome variable is very little since most of our study participants were adults (26–35 years old).

## Conclusion

The findings of our study showed that there is a higher prevalence of myopia among pregnant women in our study area. Myopia was significantly associated with maternal age, the 3rd trimester of gestation, multi & grand multiparous women, and those who had history of contraceptive use before the current pregnancy. Further prospective analytical studies regarding visual system among pregnant women, particularly as a result of pregnancy, are strongly recommended. We also recommended health professionals to perform a routine initial evaluations, promotions and preventions for the health of visual systems and pre-existing conditions of the pregnant women.

## Data Availability

The raw data supporting the conclusions of this article will be made available by the authors, without undue reservation.
